# A multi-institutional trial evaluating the use of an integrated quality assurance phantom for frameless single-isocenter multitarget stereotactic radiosurgery

**DOI:** 10.3389/fonc.2024.1445166

**Published:** 2024-10-31

**Authors:** Dante P. I. Capaldi, Lawrie B. Skinner, Daniel W. Pinkham, Sergei Zavgorodni, Olga Stafford, Maryam Shirmohammad, Jason E. Matney, Piotr Dubrowski, Paul De Jean, Elliot M. Grafil, Amy S. Yu

**Affiliations:** ^1^ Department of Radiation Oncology, University of California, San Francisco (UCSF) Comprehensive Cancer Centre, San Francisco, CA, United States; ^2^ Department of Radiation Oncology, School of Medicine, Stanford University, Stanford, CA, United States; ^3^ Department of Therapeutic Radiology, Yale School of Medicine, New Haven, CT, United States; ^4^ British Columbia Cancer Agency, Vancouver Island Centre, Victoria, BC, Canada; ^5^ Department of Radiation Oncology, Alta Bates Summit Medical Center SutterHealth, Berkeley, CA, United States; ^6^ Department of Radiation Oncology, University of Michigan, Ann Arbor, MI, United States; ^7^ Department of Radiation Oncology, University of California, Davis School of Medicine, Sacramento, CA, United States; ^8^ Luca Medical Systems, Palo Alto, CA, United States

**Keywords:** 3D printing, quality assurance, stereotactic radiosurgery, frameless brain radiosurgery treatment, off-axis Winston-Lutz, single isocenter multi-target

## Abstract

**Background:**

Brain radiosurgery treatments require multiple quality-assurance (QA) procedures to ensure accurate and precise treatment delivery of ablative doses. As single-isocenter multitarget radiosurgery treatments become more popular for treating patients with multiple brain metastases, quantifying off-axis accuracy of linear accelerators is crucial. In this study, we developed a novel brain radiosurgery integrated phantom and validated this phantom at multiple institutions to enable radiosurgery QA with a single phantom to facilitate implementation of a frameless single-isocenter, multitarget radiosurgery program. The phantom combines multiple independent verification system tests including the Winston-Lutz test, off-axis accuracy evaluation (i.e., off-axis Winston-Lutz), as well as dosimetric measurements utilizing both point dose and film measurement.

**Methods and materials:**

A novel 3D-printed phantom, coined *OneIso*, was designed with a movable insert which can switch between Winston-Lutz test targets and dose measurement without moving the phantom itself. In total, four phantoms were printed, and eight institutions participated in this study, which included both Varian TrueBeam (n=6) and Elekta Versa (n=2) linear accelerators. For off-axis Winston-Lutz measurements, a row of off-axis ball-bearings (BBs) was integrated into the OneIso. To quantify the spatial accuracy versus distance from isocenter, two-dimensional displacements were calculated between the planned and delivered BB locations relative to their respective MLC-defined field borders. For dose verification, brain radiosurgery clinical treatment plans previously treated were delivered at multiple cancer centers (six of eight centers). Radiochromic film and pinpoint ion chamber comparison measurements were obtained with OneIso.

**Results:**

Dose verification performed using the OneIso phantom across the different centers were all within on average 3% agreement, for both film and point-dose measurements. OneIso identified a reduction in spatial accuracy further away from isocenter for all eight radiosurgery machines. Differences increased as distance from isocenter increased, exceeding recommended radiosurgery accuracy tolerances (<1mm) at different distances for each machine (2-7cm), indicating that the tolerance is machine-dependent.

**Conclusion:**

OneIso provides a streamlined, single-setup workflow for single-isocenter multitarget frameless linac-based radiosurgery QA that can be easily translated to multiple institutions. Additionally, quantifying off-axis spatial discrepancies allows for determination of the maximum distance between targets and iso that meet single-isocenter multitarget radiosurgery program recommendations.

## Introduction

Over the past decade, there has been growing evidence through clinical trials supporting the role of radiosurgery for the management of patients with multiple intracranial brain metastases (>3 metastases) that would have conventionally been treated with whole-brain radiotherapy (WBRT) (1). Current workflows for treating multiple brain metastases either rely on dedicated equipment, such as the GammaKnife (Elekta, Stockholm, Sweden) or CyberKnife (Accuray, Sunnyvale, CA) radiosurgery systems, which unfortunately have extended treatment times, where the time is proportional to the number of metastases treated (2, 3). Alternatively, dedicated radiosurgery workflows on conventional C-arm linear accelerators (LINACs) have been established to handle the necessary requirements for delivering radiosurgery treatments as well as shorten the overall treatment times (4), which now represent more than half of the machines commonly used for treating brain metastasis using radiosurgery (5). Furthermore, multiple institutions have implemented single-isocenter multi-target (SIMT) radiosurgery programs on their C-arm LINACs (6), which are rapidly being deployed clinically with the assistance of vendor support, such as Varian’s HyperArc (Varian Medical Systems, Palo Alto, CA) and Brainlab’s ExacTrac (Brainlab AG, Munich, Germany) (7, 8).

A well-recognized challenge with SIMT radiosurgery treatments is the impact of rotational error on target coverage as a function of distance away from isocenter (6, 9–11). Multiple studies have not only investigated the dosimetric impact but also strategies to accommodate this uncertainty into margins (i.e., larger margins for lesions further away from isocenter) (12, 13). Furthermore, physically evaluating the mechanical alignment versus the radiation field as a function of distance away from isocenter (i.e., the off-axis Winston-Lutz test) has been incorporated into multiple quality assurance (QA) platforms (14–20) and proposed to be evaluated routinely on a monthly basis for machines delivering SIMT (15).

Accordingly, we developed a novel brain radiosurgery integrated phantom and validated this phantom at multiple institutions, on both Varian and Elekta platforms, to enable radiosurgery QA with a single phantom to facilitate the implementation of a frameless single-isocenter, multitarget radiosurgery program. The phantom combines multiple independent verification system tests including the Winston-Lutz test, off-axis accuracy evaluation (i.e., off-axis Winston-Lutz), as well as dosimetric measurements utilizing both point dose and film measurements. The purpose of this current study was to validate the previously developed and validated phantom at different institutions and observe institutional and machine specific characteristics.

## Materials and methods

### Phantom design

**Figure 1:**
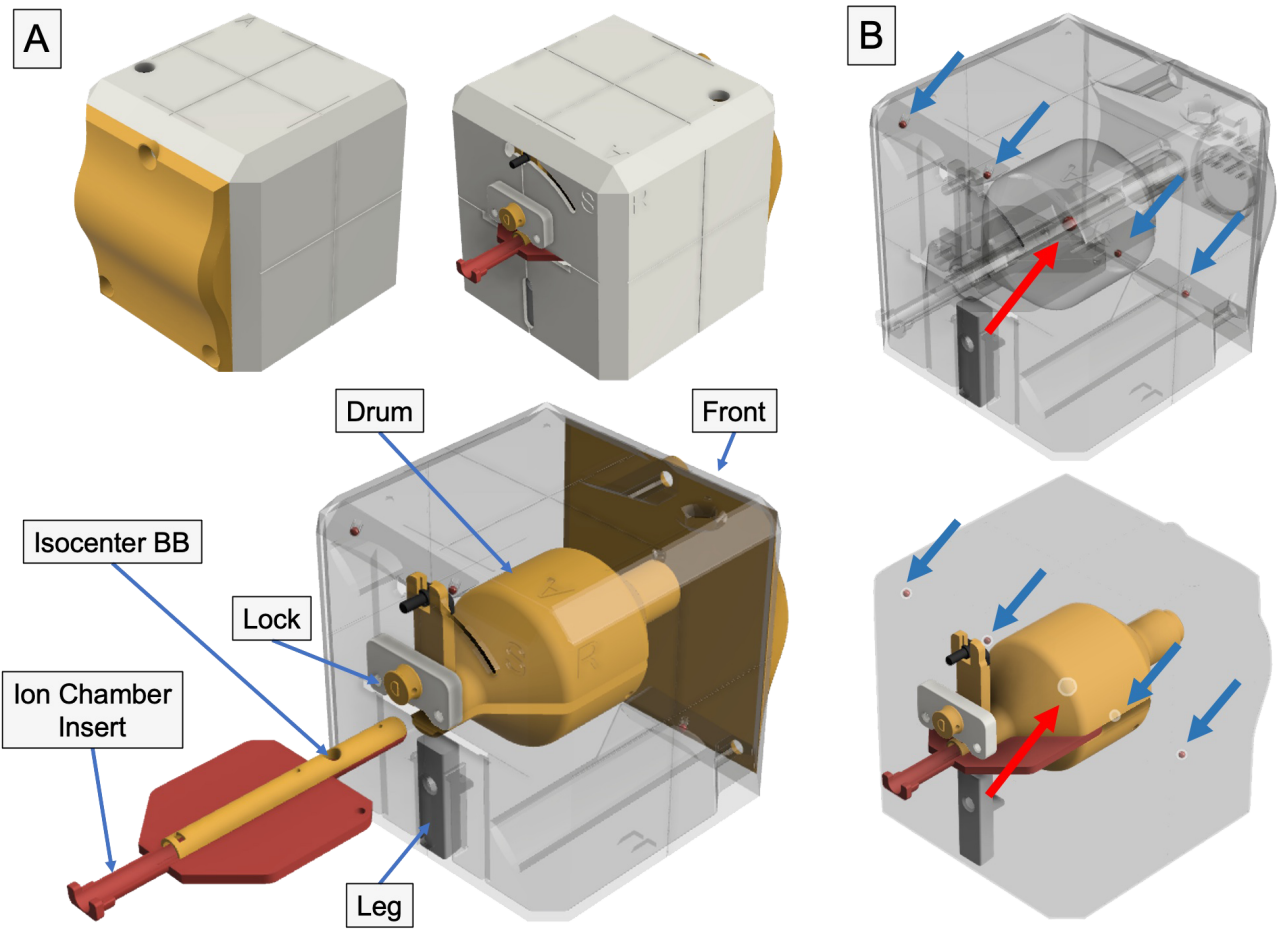
3D printed radiosurgery QA phantom – the *OneIso*. **(A)** The phantom is constructed of four major component groups and a total of twelve 3D printed parts. Notch-lock mechanisms were designed to lock the central ion chamber insert in place. Inside, there is a solid central drum. This drum contains one steel ball bearing to aid in determining the off-axis Winston-Lutz. Inside the drum, there are two independent inserts which contain the isocenter ball bearing as well as either an ion chamber (shown above) or film inserts. **(B)** The phantom contains a row of off-axis ball bearings designed to have no overlap with any ball bearings in the anterior-posterior and lateral directions to quantify the spatial accuracy of a linear accelerator-based single-isocenter, multitarget cranial radiosurgery system.

We have previously designed and developed a phantom (21), coined OneIso, using Fusion 360 (Autodesk, San Rafael, CA) as depicted in **Figure 1** and **Supplementary Video 1**. This phantom was designed to provide independent verification of each positioning system and coincidence of each system (kV, MV, CBCT, optical surface imaging, Winston–Lutz test, off-axis Winston-Lutz test, light/radiation field coincidence, and lasers), all integrated into OneIso. The phantoms were printed with polylactic acid (PLA) plastic at a density of 1.15 - 1.2 g/cc, specifically an Ultimaker S5 (Ultimaker, Cambridge, MA) 3D printer. The ball bearings (BBs) included in the phantom design has one 6 mm diameter central tungsten ball bearing for Winston-Lutz tests and a row of four off-axis 3 mm diameter steel BBs for determining off-axis Winston-Lutz test. In this study, we primarily focused on evaluating the off-axis Winston-Lutz test and radiosurgery patient-specific dosimetric measurements for centers that were currently performing radiosurgery on their C-arm LINACs (if applicable).

Within the OneIso, there is a solid central drum that facilitates not only the Winston-Lutz tests (with a central BB) but also dosimetric testing (both ion chamber and film measurements). Couch index bar mounts were also designed and printed for both the Varian Exact bar (Varian Medical Systems, Palo Alto, CA) as well as the Elekta iBEAM indexing bar (Elekta, Stockholm, Sweden) for easy setup (the couch can be sent to the predefined position).

### Off-axis Winston Lutz analysis

**Figure 2:**
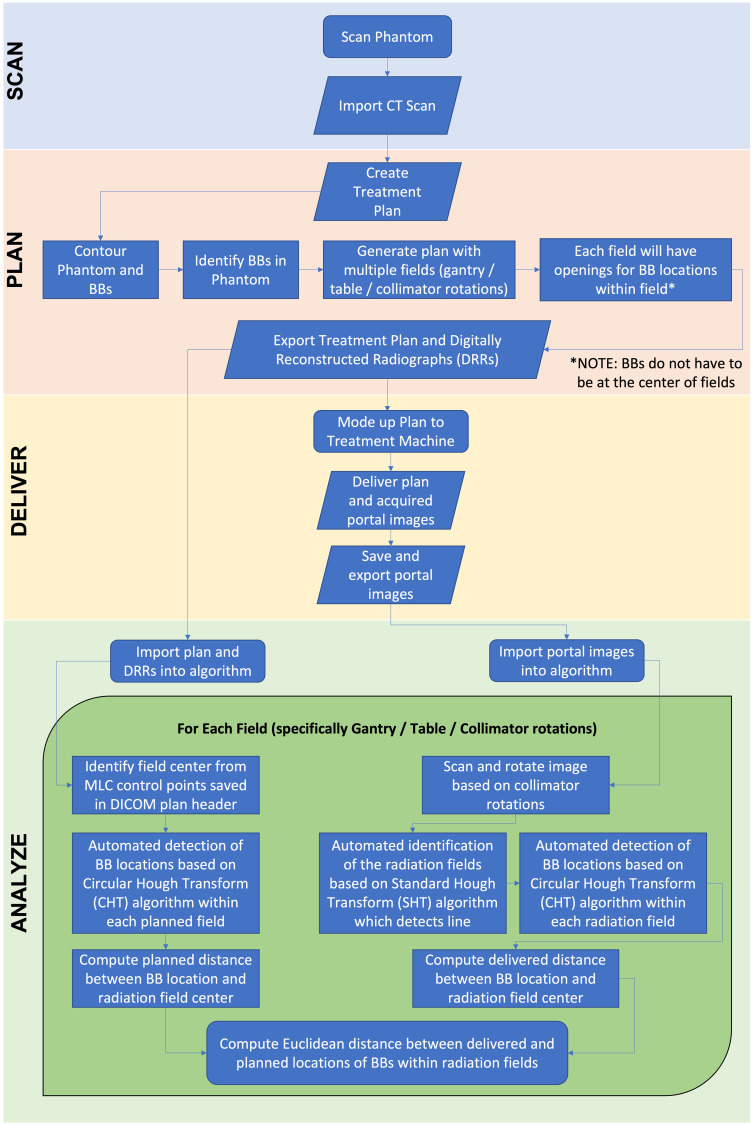
Flowchart of off-axis Winston-Lutz test. The flowchart is broken down into four distinct parts: *Scan, Plan, Deliver, Analyze*. The phantom is scanned and imported into the treatment planning system. An off-axis Winston-Lutz treatment plan is created (based on the pre-created plans that can be downloaded from GitHub) for delivery. Once delivered, the DRRs are compared to the acquired portal images to determine the difference in distances.

**Figure 2** shows the flowchart describing the process of analyzing the off-axis Winston-Lutz test. Specifically, the test is broken down into each of the following components: Scan, Plan, Deliver, and Analyze. For scanning, a computed tomography (CT) scanner is used to acquire the 3D image of the phantom with the five BBs used for the off-axis Winston-Lutz test, where the CT digital Imaging and Communications in Medicine (DICOM) data of the QA phantom is imported into the treatment planning system. The treatment planning system is used to create the plan that will be delivered at the machine, where the phantom, as well as BBs, are segmented/contoured to volumetrically determine the locations of the BBs. The contoured images of the phantom and the BBs are then used to identify the BBs and their relationship to the center of the phantom (set at the isocenter of the treatment machine). The now contoured CT scan of the phantom is used to create fields (radiation beams and their specific geometry) with different collimator, table, and gantry rotations (analogous to the traditional Winston-Lutz test that samples only the center of the treatment space – isocenter). The following are the different gantry (G), collimator (C), and table (T) combinations we implemented: C1: G0°/C90°/T0°, C2: G0°/C45°/T0°, C3: G0°/C315°/T0°, C4: G0°/C270°/T0°, G1: G270°/C0°/T0°, G2: G0°/C0°/T0°, G3: G90°/C0°/T0°, G4: G180°/C0°/T0°, T1: G0°/C0°/T90°, T2: G0°/C0°/T45°, T3: G0°/C0°/T315°, T4: G0°/C0°/T270°.

For each of the above fields, the multi-leaf collimator (MLC), which is a beam-shaping device, is used to shape the radiation field to have five distinct apertures in a single field for each of the BBs identified and contoured from the imported CT scan. One important thing to note is that the BBs relative to the apertures do not have to be in the center of the fields (compared to other methods – i.e., traditional Winston-Lutz test requires the BB to be in the center of the field). Alternatively, we use the treatment plan (location of the MLC) and BB locations as *a priori* information for comparison. Following the treatment planning step, Digitally Reconstructed Radiographs (DRRs) are produced and exported from the treatment planning system. For DRR generation (for optimal software analysis) the parameters of the DRR were manually adjusted to have the CT HU data between 100 and 1000HU. Specifically, the focus was on producing good quality images for analysis; showing only BBs demarked as clear circles without other parts of the phantom. The physical distances of the BBs are known by the design of the phantom, albeit the fabrication process incorporates slight variations across the different phantoms. Here, we leverage reference CT DRRs to identify the BBs.

**Figure 3:**
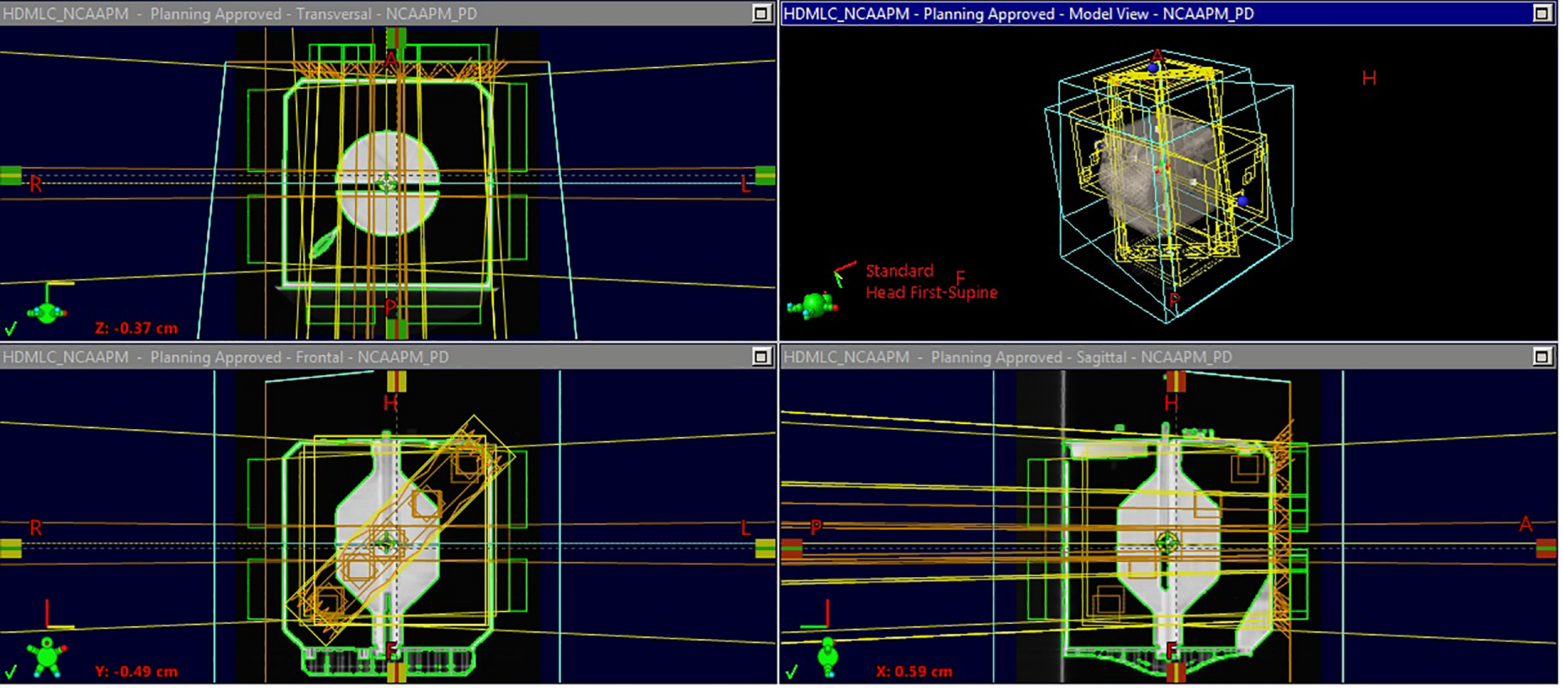
CT scan of OneIso and Off-axis Winston-Lutz beam geometry.

**Figure 4:**
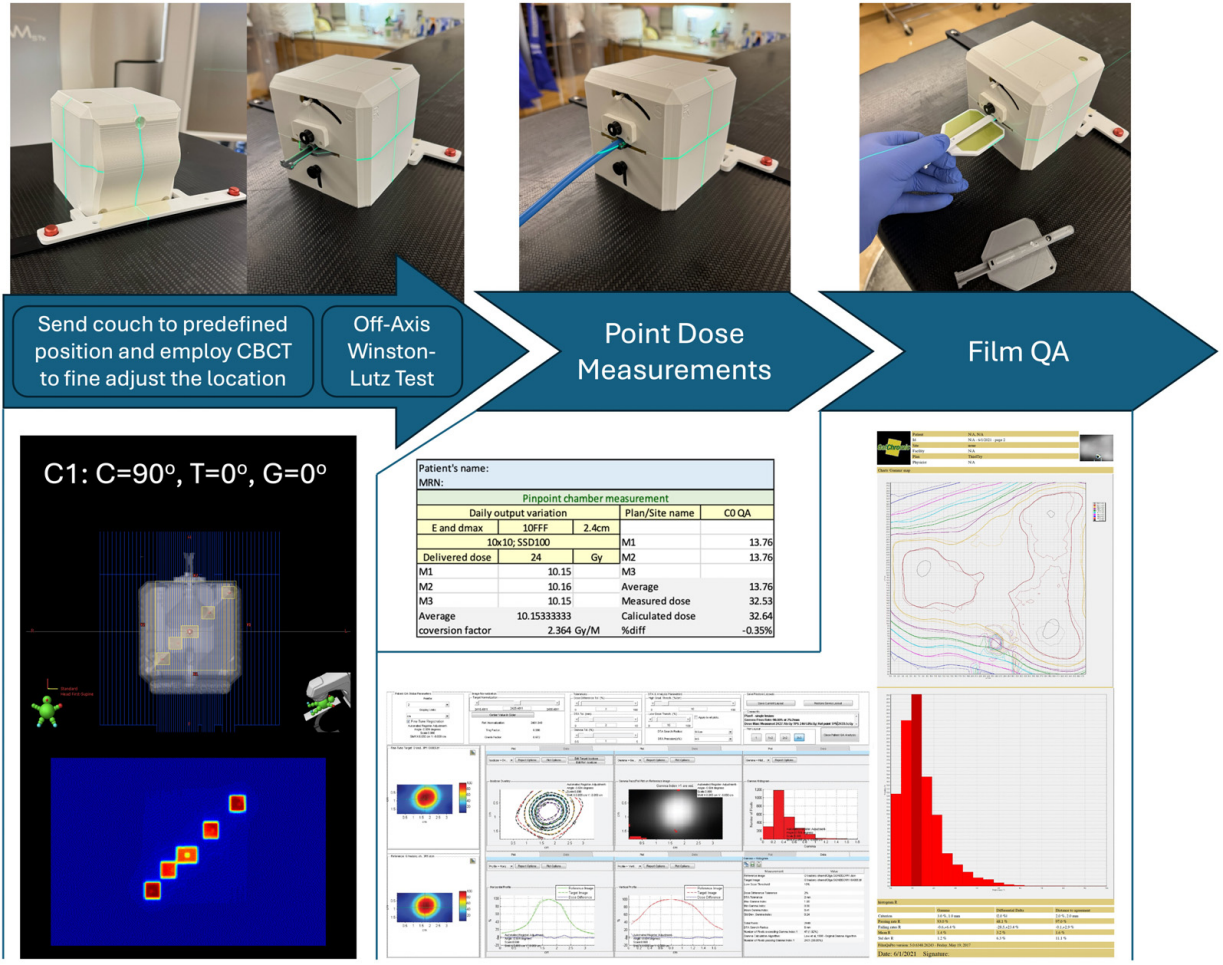
OneIso workflow on the machine for acquiring Off-axis Winston-Lutz and dosimetric QA measurements. First, the OneIso is set up on the couch and set to predefined position. CBCT is acquired and aligned to reference CT to finetune the location. Off-axis Winston-Lutz test is then acquired. Dosimetrically, both point base measurements as well as film measurements for patient specific QA are acquired. Film analysis is performed using current clinical film QA workflows.

The generated treatment plan, containing radiation fields with all their associated parameters and movement commands are imported into the LINAC. With the OneIso phantom present, the radiation fields are then delivered. During this process the Electronic Portal Imaging Device (EPID) is acquiring portal images for all radiation fields. The recorded portal images are then exported to be used in the analysis phase. **Figures 3** and **4** illustrate the OAWL beam geometry and workflow on the machine for acquiring OAWL as well as dosimetric QA measurements.

**Figure 5:**
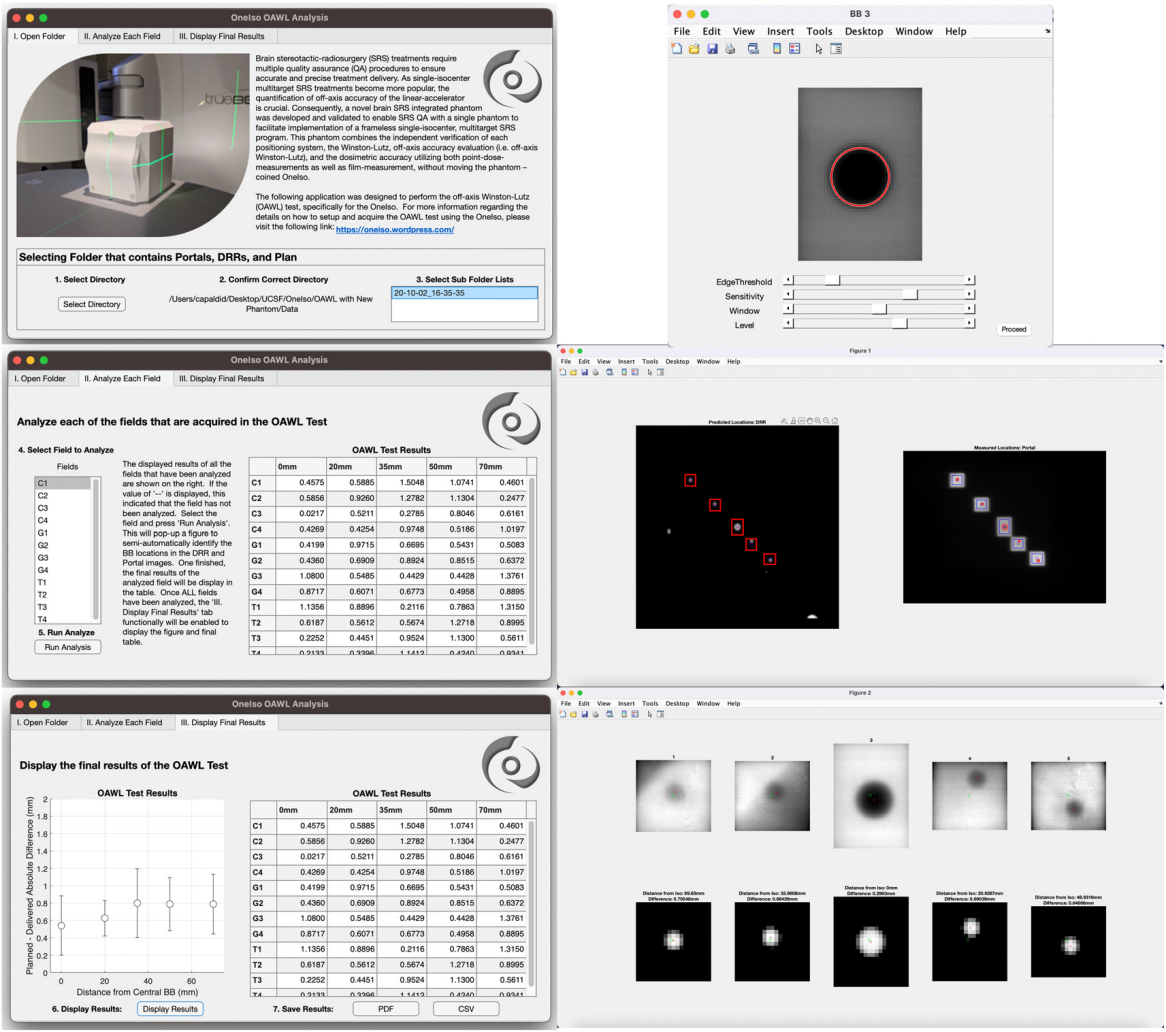
Off-axis Winston-Lutz analysis application: A MATLAB script was developed to analyze the images acquired from each cancer center to calculate the results of the off-axis Winston-Lutz test.

As shown in **Figure 5**, an analysis software package was developed in MATLAB R2022b (MathWorks, Natick, MA) which quantifies the spatial discrepancy as a function of distance from isocenter by calculating the absolute differences in the planned versus the delivered BB locations, as described above. A video demonstrating the application is in the **Supplementary Video 2**.

For analysis, first the treatment plan and DRRs produced from the treatment planning system are exported and imported into the analysis software. From the exported DICOM treatment plan, the MLC control points (i.e., the locations of the MLCs) for each field were saved and identified in the DICOM header of the treatment plan file. The locations of the MLCs were used to generate each specific aperture for each BB (i.e., 5 BBs with 5 apertures for each field {gantry, collimator, and table rotation}). The centers of the aperture were determined from the midpoint between the two corners of the aperture. The cropped DRR images are then used to perform automated detection of BB locations based on the Circular Hough Transform (CHT) algorithm (22, 23). This algorithm determines the centers of each BB for each aperture (one BB center for each of the 5 fields). From this, the distance (both magnitude and direction) between each BB center location and each aperture center location generated from the MLC for each radiation field was determined.

Next, the acquired portal images from the treatment delivery machine are imported into the developed in-house analysis software. The imported portal images were rotated in the software based on the collimator rotations to correct the alignment between the DRRs (planned) and the portal (acquired) images for comparison. The rotated portal images are then used to perform automated detection of BB locations based on the Standard Hough Transform (SHT) algorithm (24–26). Similar to the DRR analysis, the developed algorithm determines the centers of the fields for each aperture created from the MLCs from the portal images. The apertures that are identified (5 apertures in total) are then cropped from the portal images. Similar to the DRRs, the cropped and rotated portal images are then used to perform automated detection of BB locations based on the CHT algorithm. This algorithm determines the centers of each BB for each aperture (one BB center for each of the 5 aperture). Once identified, the distance (both magnitude and direction) between each BB center location and each aperture center location generated from the MLC for each radiation field were determined. Finally, the distances computed from the treatment plan and the portal images acquired during delivery are then used to compute the Euclidean distance between delivered and planned locations of BBs within radiation fields; ultimately identifying the magnitude of the error in the BB locations within the delivered radiation fields as compared to the planned locations. These steps outlined above are repeated for each gantry, collimator, and table position. Once all fields have been analyzed, the computed error for each BB for each radiation field as a function of distance away from machine isocenter is aggregated into a plot as illustrated in the results.

### Ion chamber and film dosimetry

Dosimetric data were acquired according to each institutional protocol, using calibrated ion chamber and film methods. Each institution was asked, in addition to the OAWL test, to perform patient-specific QA for radiosurgery patients if patients were previously treated with a C-arm LINAC using the OneIso (either using film, ion chamber, or both). Information about the plans delivered at each institution is shown in **Table 1**. Film was cut to fit the film insert and placed within the drum of the OneIso, and film analysis was performed using center specific protocols and software (FilmQA Pro and RIT software). Ion chamber measurements were performed using a pinpoint ion chamber placed in the ion chamber insert.

**Table 1:** Description of the multiple centers participating in this trial and patient-specific plans used to perform the dose verification.

	Institutional Machine and Plan Information				
	*Machines/MLCs*	*No. Targets*	*Rx Dose (Gy)*	*Beam Quality (MV)*	*Arcs/Geometry*
*Center 1*	Varian/HD-MLC	1/1/1	20/24/18	10FFF/10FFF/10FFF	2/Coplanar
*Center 2*	Varian/HD-MLC	1/1/1	20/24/18	10FFF/10FFF/10FFF	2/Coplanar
*Center 3*	Varian/HD-MLC	1/1/1	20/24/18	10FFF/10FFF/10FFF	2/Coplanar
*Center 4*	Elekta/Agility	3	20	6FFF	3/Coplanar
*Center 5*	Varian/HD-MLC	––	––	––	––
*Center 6*	Varian/HD-MLC	1/2	20/20	6X/6FFF	4/3/Non- coplanar
*Center 7*	Varian/HD-MLC	1/1/1	22/20/22	6FFF/6FFF/6FFF	3/3/3/Non- coplanar
*Center 8*	Elekta/Agility	––	––	––	––

### Testing centers

Multiple phantoms were printed, manufactured, and shipped to various institutions from June 2020 to May 2023, where data was acquired at eight clinics. Clinics were asked to acquire the off-axis Winston-Lutz data, as well as any dosimetric data on patient-specific radiosurgery plans delivered on their C-arm linacs. As shown in **Table 1**, six of the eight clinics used Varian TrueBeams (Varian Medical Systems, Palo Alto, CA), while the other two centers used Elekta Versas (Elekta, Stockholm, Sweden).

## Results

### Off-axis Winston-Lutz test

**Figure 6:**
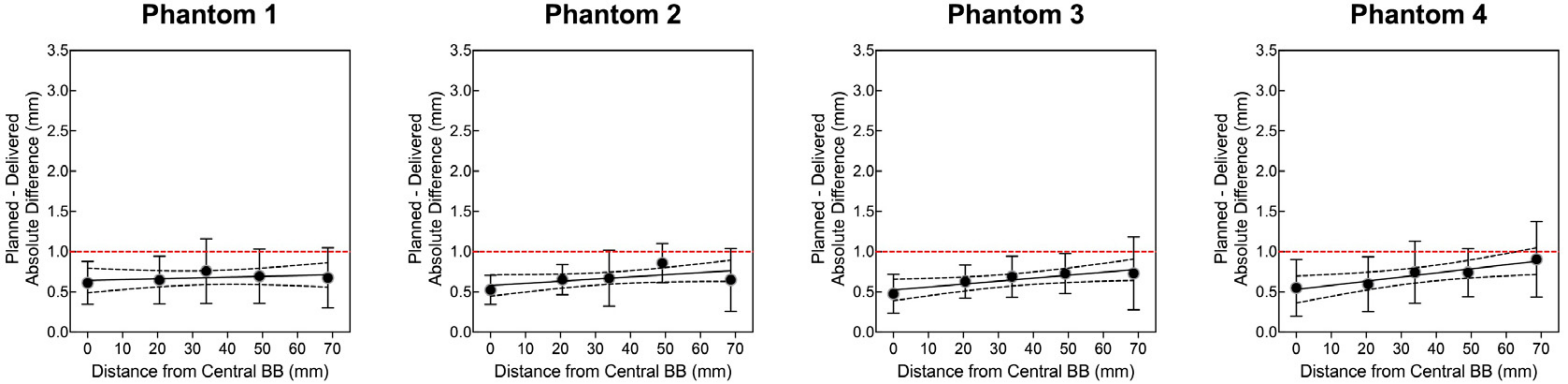
The quantitative Winston-Lutz 2D displacements of the off-axis BB locations for the four different phantoms manufactured and sent to the different institutions. Points represent the mean, error bars represent the standard deviation, a line represents the linear regression, dotted lines represent the 95% confidence interval, and a red dashed line represents the 1 mm Winston-Lutz threshold.

**Figure 7:**
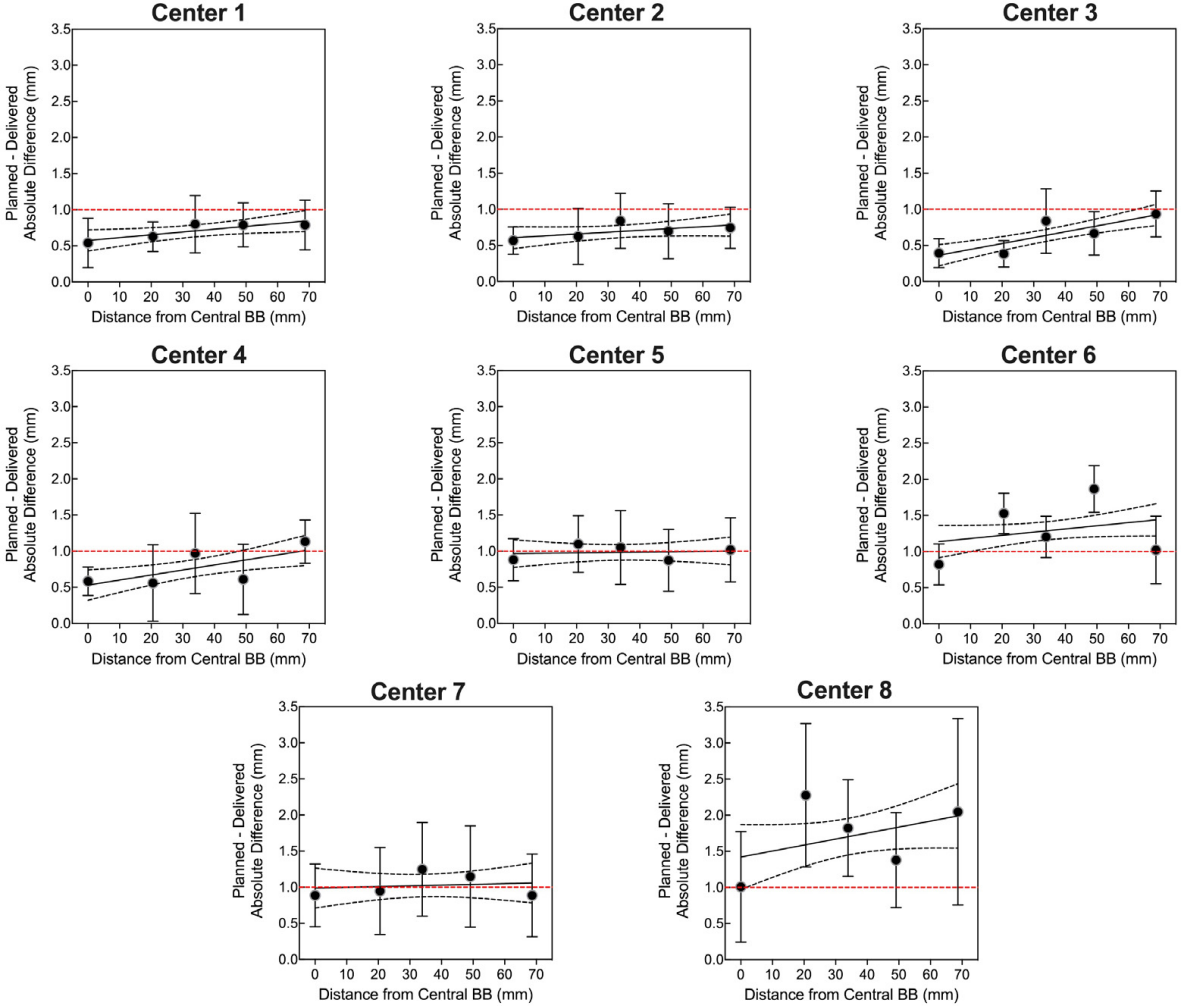
The quantitative Winston-Lutz 2D displacements for off-axis BB locations at the eight different centers. Points represent the mean, error bars represent the standard deviation, a line represents the linear regression, dotted lines represent the 95% confidence interval, and a red dashed line represents the 1 mm Winston-Lutz threshold.

Four OneIso phantoms were printed and sent to multiple institutions. **Figure 6** shows the OAWL test for all four phantoms performed on the same C-arm LINAC (Varian TrueBeam) - these four phantoms had minimal variations when tested on the same machine (repeated measures ANOVA, p=0.6). **Figure 7** shows the spatial accuracy as a function of distance from the isocenter for all eight cancer centers, illustrating that the spatial accuracy reduces further away from the isocenter. Differences increased as the distance from the isocenter increased, exceeding recommended radiosurgery accuracy tolerances at 2-7 cm away from the isocenter. As illustrated in **Figure 7**, all eight radiosurgery machines exceeded the recommended accuracy tolerance at different distances away from the isocenter, suggesting this measurement is machine dependent.

### Ion chamber and film dosimetry

The percent dose difference between the treatment planning system (Dose_TPS_) and the pinpoint ion chamber measurements in the OneIso phantom (Dose_OneIso_) for the multiple institutional patient-specific treatment verification plans are listed in **Table 2**. The average percent dose difference between Dose_OneIso_ and Dose_TPS_ was 0.2 ± 0.7%. The gamma passing rate based on the measured dose compared to the calculated dose exported from the treatment planning system using each institutional passing criteria for OneIso are listed in **Table 3**. The gamma passing rate for OneIso was on average 97.5 ± 1.5%. The difference between doses were less than 3% across all participating cancer centers.

**Table 2:** The percent dose difference (Δ) between the dose measured by ion chamber in the OneIso phantom (D_OneIso_) and dose calculated by the treatment planning system (D_TPS_).

	Δ(D_OneIso_ – D_TPS_)%^†^				
	*Plan 1*	*Plan 2*	*Plan 3*	*Average (SD)*	
*Center 1*	-0.7%	1.2%	-1.2%	-0.2 (1.3) %	
*Center 2*	-1.6%	-0.3%	-0.7%	-0.8 (0.7) %	
*Center 3*	1.8%	1.7%	1.5%	1.7 (0.2) %	
*Center 4*	––	––	––	––	
*Center 5*	––	––	––	––	
*Center 6*	––	––	––	––	
*Center 7*	––	––	––	––	
*Center 8*	––	––	––	––	
*Average (SD):*				0.2 (0.7) %	

**^†^** Pinpoint ion-chamber measurements in OneIso phantom (D_OneIso_) and the dose calculated by the treatment planning system (D_TPS_).

**Table 3:** The gamma passing rate (g) between the dose measured by film in the OneIso phantom, and the dose calculated by the treatment planning system.

	g(Film_OneIso_)%^†^				
	*Plan 1*	*Plan 2*	*Plan 3*	*Average (SD)*	
*Center 1*	100.0%	94.2%	99.2%	97.8 (3.1) %	
*Center 2*	100.0%	98.5%	98.8%	99.1 (0.7) %	
*Center 3*	100.0%	95.0%	98.2%	97.7 (2.5) %	
*Center 4*	93.0%	––	––	93.0 (–) %	
*Center 5*	––	––	––	––	
*Center 6* ^††^	98.1%	96.6%	––	97.4 (1.0) %	
*Center 7* ^†††^	99.8%	100.0%	99.9%	99.9 (0.1) %	
*Center 8*	––	––	––	––	
*Average (SD):*				97.5 (1.5) %	

^†^Film measurements in OneIso phantom (Film_OneIso_) compared with the dose distribution calculated by the treatment planning system (3%/1mm, 10% threshold). ^††^2%/2mm, 10% threshold. **^†††^** 4%/1mm, 10% threshold.

## Discussion

Radiosurgery treatment delivers a very high dose per fraction (i.e., ~16 – 24 Gy in a single fraction for brain metastases), and the margin of the planning target volume (PTV) is very small compared to conventional radiotherapy. Therefore, special attention and diligence are required before a radiosurgery program gets implemented clinically. Moreover, the target size is usually very small (i.e., < 1 cc), thus a small error in target localization will result in risks of undertreatment of portions of the tumor by 20% or more and overdosage of adjacent normal tissues (10). It could escalate the risk of serious injury to a much greater degree than an equivalent treatment error in a course of radiotherapy where a substantially lower dose per fraction is used (27).

In practice, the success of radiosurgery treatment depends critically on the proper commissioning of the radiosurgery delivery machine. Adequate confidence that a radiosurgery machine will satisfy the clinical requirements for quality is achieved by a QA program consisting of a series of planned and systematic actions. Two American Association of Physicists in Medicine (AAPM) recommendations have highlighted the importance of routine QA and provided comprehensive descriptions of important tests that must be performed at regular intervals and tolerances (27, 28). Radiosurgery QA provides the confidence that planned dose will be accurately delivered to the patient with consideration of the spatial accuracy of the machine and all the imaging modalities used during treatment. By knowing the uncertainties and errors in dosimetry, equipment performance, and treatment delivery, QA becomes an integral part of a viable radiosurgery program. High dosimetric and geometric accuracy is a prerequisite for comprehensive tumor control as well as playing a key role in reducing the likelihood of complications, accidents, and medical events. High-quality, consistent QA also allows reliable intercomparison of results between different radiotherapy centers, ensuring a more uniform and accurate treatment delivery. This is necessary for clinical trials and sharing clinical radiotherapy experience between centers (27, 29, 30).

In this study, a multi-institutional evaluation was conducted in an effort to translate this technology to all cancer centers and to collect images to further optimize the imaging analysis. It is essential to know the performance of each LINAC before clinical radiosurgery treatment implementation. The recommended radiosurgery accuracy tolerance is 1 mm overall, including couch, gantry, collimator, and MLC, with the proper QA procedure to determine the spatial delivery accuracy as a function of distance from the isocenter being crucial for the single-isocenter, multitarget radiosurgery program (27). Through the multi-institutional evaluation, we showed that: 1) the use of the proposed integrated QA phantom provides the ability to quantify the off-axis spatial discrepancies; 2) the system is capable of providing dosimetric information needed for patient-specific QA; and 3) the implementation of this new paradigm of QA will significantly improve radiosurgery treatment QA workflow via a single phantom setup with comprehensive data collection and analysis. A custom mount was also designed to directly integrate with the Varian HyperArc system, which has been deployed to deliver noncoplanar radiosurgery treatments. Software was developed and optimized for: 1) varying image quality, which might be produced by different users/machines; and 2) semi-automatic data processing for analysis of the acquired images. A novel strategy and application of feature extraction techniques were used to analyze the images so we can take advantage of the DRRs and the treatment planning DICOM files to automatically identify the prescribed radiation field as well as the contoured BB locations. The image processing pipeline was optimized for the quantification of off-axis spatial accuracy. This method eliminated the need for the hidden target to be at the center of the radiation field and allowed any combination of beam arrangement for the QA process. In particular, we use the DRRs that are generated from the reference CT, and using the reference CT to align the phantom to the CBCT acquired on the couch before delivering the OAWL test. Inherently, this relies on the imaging and radiation isocenters to be calibrated, which is common practice for modern C-arm linacs (i.e., the IsoCal test by Varian), and evaluated on a periodic basis as the majority of treatments rely on image guidance. The method proposed here for evaluating OAWL aligns with how we currently treat patients on C-arm linacs, providing a more “End-to-End” evaluation of the targeting accuracy based on the reference/planning CT. Furthermore, the use of EPID imaging is commonly used for performing WL tests, which was also leveraged here in this study (31). Additionally, we incorporated five BBs into the previously designed phantom (21) in a line, as illustrated in **Figure 1**. By adding more BBs, across the whole phantom, this would facilitate a more thorough evaluation of the OAWL test, albeit the phantom was designed and optimized for 3D printing by minimizing overhangs in any single piece and incorporating more BBs could compromise the design.

In addition to the data acquired at each institution, we also observed other practical considerations in this multi-institutional study. First, in the process of evaluating this phantom design at multiple institutions, we fabricated four 3D printed phantoms, which were ultimately shipped out and shared at the participating centers. Prior to deploying these phantoms at the different clinics, we first evaluated them on one machine, to determine if there were any variations in the manufacturing process (as illustrated in **Figure 6**), which we observed to be minimal. Furthermore, we requested acquisition of the OAWL test as well as any radiosurgery patient-specific dosimetric data at each of the participating institutions, if they currently perform radiosurgery treatments on their C-arm LINACs. We observed that six of the eight cancer centers perform LINAC radiosurgery treatments, where the majority performed film QA to evaluate patient-specific QA. Film analysis criteria was decided by each of the participating centers based on their own institutional criteria, as this phantom was designed to integrate into the current clinical QA workflows. A limitation that was observed when deploying the phantom was that it was designed to only hold a Pinpoint chamber (PTW Model N31014, Frieburg, Germany), limiting the use of this current phantom design to institutions that have this specific chamber. We have further developed modifications to the ion chamber insert to be flexible with the choice of ion chamber used to acquire dosimetric data.

In conclusion, we developed an integrated QA phantom which streamlines the necessary QA for single-isocenter multitarget frameless linac-based radiosurgery programs into a single setup workflow in an effort to easily translated this phantom to multiple institutions. The phantom was evaluated at multiple institutions and demonstrated the off-axis spatial discrepancies from isocenter for each individual machine, indicating that need for machine-specific evaluation. Accordingly, the results of this study support the recommendation for off-axis Winston Lutz testing to be performed on a routine basis for machines delivering SIMT.

## Data Availability

The raw data supporting the conclusions of this article will be made available by the authors, without undue reservation.
